# Phylogeny of *Vietnamosasa* (Poaceae, Bambusoideae) based on syntenic nuclear genes with description of a new species

**DOI:** 10.3897/phytokeys.273.182677

**Published:** 2026-04-14

**Authors:** Xiao Feng, Zu-Chang Xu, Jing-Xia Liu, Mei Chen, Dmitry D. Sokoloff, Constantin I. Fomichev, Meng-Yuan Zhou, De-Zhu Li

**Affiliations:** 1 Germplasm Bank of Wild Species & Yunnan Key Laboratory of Crop Wild Relatives Omics, Kunming Institute of Botany, Chinese Academy of Sciences, Kunming, Yunnan 650201, China Yunnan University Kunming China; 2 University of Chinese Academy of Sciences, Beijing 100049, China M.V. Lomonosov Moscow State University Moscow Russia; 3 Institute of Biodiversity, School of Ecology and Environmental Science, Yunnan University, Kunming 650504 Yunnan, China Kunming Institute of Botany, Chinese Academy of Sciences Kunming China; 4 School of Plant Sciences and Food Security, Faculty of Life Sciences, Tel Aviv University, Tel Aviv 6997801, Israel Shandong Agricultural University Shandong China; 5 Faculty of Biology, M.V. Lomonosov Moscow State University, 1, 12, Leninskie Gory, 119234 Moscow, Russia Tel Aviv University Tel Aviv Israel; 6 Center for Interdisciplinary Biodiversity Research & College of Forestry, Shandong Agricultural University, Tai’an, Shandong 271018, China University of Chinese Academy of Sciences Beijing China

**Keywords:** BDG complex, deep genome skimming, syntenic nuclear genes, *
Pseudobambusa
*, *Vietnamosasa sakonnakhonensis*

## Abstract

*Vietnamosasa* is a small paleotropical woody bamboo genus of the *Bambusa*-*Dendrocalamus*-*Gigantochloa* (BDG) complex with unresolved interspecific relationships. It grows in the dry dipterocarp forest and adapts to seasonal dryness and annual burning. Here, we reconstructed the phylogeny of *Vietnamosasa* using 938 syntenic nuclear genes. Our phylogenetic analysis based on four nuclear gene datasets from three subgenomes, i.e., subgenomes A, B, C, and all subgenomes combined, revealed that all species of *Vietnamosasa*, including a putative new species, clustered as a monophyletic group with high support, and *Pseudobambusa* is closely related to *Vietnamosasa*. Within *Vietnamosasa*, *V. pusilla* and *V. darlacensis* form a monophyletic group sister to *V. ciliata*. However, extensive discordance among gene trees indicates the complex reticulate evolution within *Vietnamosasa*. On the basis of morphological comparisons with the three published species of *Vietnamosasa*, together with phylogenomic evidence, we described a new species, *V. sakonnakhonensis* from Thailand, and provided a key to the four recognized species. The new species is distinguished from other species of *Vietnamosasa* by its wider foliage leaf blade and longer pistil.

## Introduction

Paleotropical woody bamboos (PWB) are widely distributed in Asia, Africa, and Oceania. They are currently divided into eight subtribes: Melocanninae Benth., Racemobambosinae Stapleton, Temburongiinae K.M.Wong, Greslaniinae K.M.Wong & W.L.Goh, Holttumochloinae K.M.Wong & W.L.Goh, Dinochloinae K.M.Wong & W.L.Goh, Hickeliinae A.Camus, and Bambusinae J.Presl (Soreng et al. 2022). The *Bambusa*-*Dendrocalamus*-*Gigantochloa* (BDG) complex is the most intractable within the subtribe Bambusinae and is a major part of the core Bambusinae. As a highly diverse group consisting of four major genera (*Bambusa* Schreb., *Dendrocalamus* Nees, *Gigantochloa* Kurz ex Munro, and *Melocalamus* Benth.) and seven small genera (*Maclurochloa* K.M.Wong, *Oreobambos* K.Schum, *Oxytenanthera* Munro, *Pseudobambusa* T.Q.Nguyen, *Pseudoxytenanthera* Soderstr. & R.P.Ellis, *Thyrsostachys* Gamble, and *Vietnamosasa* T.Q.Nguyen), the BDG complex has attracted considerable attention in phylogenetic studies (Goh et al. 2013; Zhou et al. 2017; Liu et al. 2020; Liu et al. 2024). The majority of phylogenetic research, however, has concentrated on the four major genera, i.e., *Bambusa*, *Dendrocalamus*, *Gigantochloa*, and *Melocalamus*, with a few phylogenetic studies involving other smaller genera (Liu et al. 2020, 2023; Chen et al. 2025a, 2025b). To gain a comprehensive understanding and ascertain the relationships within the BDG complex, it is irreplaceable to cover other genera, such as *Vietnamosasa*, using syntenic nuclear genes.

The southeastern Asian bamboo genus *Vietnamosasa* is a component of the dry deciduous dipterocarp forest across Vietnam, Laos, Thailand, and Cambodia (De Bels et al. 2021). The genus is distinguished from the other genera in the BDG complex by long lanceolate foliage leaves and the subterranean basal culm (Nguyen 1990; Haevermans et al. 2013). Additionally, the above-ground culms exhibit two distinct character states: during the dry season, the culms are leafless, whereas during the rainy season, the branches develop a bush-like appearance (Dransfield 2000).

The genus *Vietnamosasa* was established by Nguyen (1990), who designated *V. darlacensis* T.Q.Nguyen as the type. It is a small genus with three recognized species (Nguyen 1990). Apart from its type, the other two species are *V. ciliata* (A.Camus) T.Q.Nguyen and *V. pusilla* (A.Chev. & A.Camus) T.Q.Nguyen. The three species are similar to one another in morphology, thus difficult to distinguish based solely on morphological characteristics. For instance, *V. darlacensis* is different from *V. ciliata* in the shape of the palea apex, while *V. ciliata* and *V. pusilla* differ in the spikelet length and the color of young shoots (Nguyen 1990; Dransfield 2000). Moreover, the generic status of *V. pusilla* and *V. ciliata* was controversial until *Vietnamosasa* was established. Both of them were first published under the temperate bamboo genus *Arundinaria* Michx. (Camus 1919; Chevalier and Camus 1921). Later, *V. ciliata* was transferred to the PWB genus *Oreiostachys* (a synonym of *Nastus* Juss.) as *O. ciliata* (Nakai 1925). Sixty or more years later, both species were transferred to *Racemobambos* Holttum, and *V. pusilla* was considered a synonym of *V. ciliata* (Chao and Renvoize 1989). It was not until 1990 that Nguyen established *Vietnamosasa* and accommodated the two species. Nevertheless, before the molecular phylogenetic analysis, the debate over whether *Vietnamosasa* should be recognized as a distinct genus or belong to *Racemobambos* had persisted (Stapleton et al. 1997). Molecular investigations eventually clarified its phylogenetic position within the Bambusoideae. Using five plastid fragments, Sungkaew et al. (2009) suggested that *Vietnamosasa* belongs to Bambusinae rather than Racemobambosinae. This complicated taxonomic history was resolved through the nomenclatural and typification clarifications provided in Haevermans et al. (2013). Subsequent studies further confirmed that *Vietnamosasa* should be placed in the core Bambusinae using several plastid and nuclear loci (Goh et al. 2013; Zhou et al. 2017). Liu et al. (2024) utilized *Mi*ddRAD data to demonstrate that *Vietnamosasa* is a monophyletic genus embedded in the BDG complex with high bootstrap support. However, relationships and morphological comparisons among the published species remain unresolved.

During our field survey in Thailand in 2018, one species with a “grass-mound” appearance was collected by the roadside near the Phu Phan National Park, Sakon Nakhon province. Through comprehensive morphological observation and comparison, it is suggested that this species is a member of *Vietnamosasa* with its long and narrow foliage leaves. It can be distinguished from other species of the genus by its longer and broader foliage leaves and glabrous culm leaf auricles. As a result, we propose to recognize it as a new species of *Vietnamosasa*. Moreover, given the lack of detailed morphological descriptions for the type of this genus and the fact that the other two species are morphologically similar, it is crucial to employ a phylogenomic approach based on syntenic nuclear genes to confirm the status of the new species and explore the relationships among the species.

With emerging studies based on deep genome skimming (DGS), it is feasible to retrieve large numbers of single-copy nuclear genes (Liu et al. 2021). For bamboos, the syntenic genes, especially the “perfect-copy” syntenic genes (as defined by Guo et al. 2019), with strict criteria matching their ploidy, have revealed the complex reticulate evolution history of woody bamboos (Ma et al. 2024). Using syntenic nuclear genes retrieved from DGS data, the hybrid origin of *Pseudosasa gracilis* S.L.Chen & G.Y.Sheng is confirmed (Hu et al. 2024), and the phylogenetic positions of two new species in *Melocalamus* are also determined (Chen et al. 2025a, 2025b). In this study, we integrate morphological comparisons with phylogenomic analysis based on syntenic nuclear genes recovered from DGS data to comprehensively study the interspecific relationships of *Vietnamosasa* with the description of a new species.

## Materials and methods

### Morphological observation

A sample of the new species was collected from Na Mong, Sakon Nakhon, Thailand, in June 2018. The species’ morphological characteristics, especially the features of spikelets, florets, foliage leaves, and culm leaves, were compared to those of *V. pusilla*, *V. ciliata*, and *V. darlacensis* based on field observations, photos, specimens, and related literature (Nguyen 1990; Dransfield 2000). We examined voucher specimens, particularly type specimens housed in the herbarium of Kunming Institute of Botany, Chinese Academy of Sciences, Kunming, Yunnan, China (KUN), as well as digital images of specimens from the Muséum National d’Histoire Naturelle, Paris, France (P).

### Taxon sampling for phylogenetic analysis

Based on previous studies (Zhou et al. 2017; Liu et al. 2024), we selected a total of 32 individuals of 22 species representing 11 genera of Bambusinae. The ingroups included six genera of the BDG complex, i.e., *Bambusa* (3 samples), *Dendrocalamus* (2 samples), *Gigantochloa* (1 sample), *Melocalamus* (2 samples), *Pseudobambusa* (1 sample), and *Vietnamosasa* (13 samples). Five other genera of subtribe Bambusinae, i.e., *Bonia* Balansa (3 samples), *Laobambos* Haev., Lamxay & D.Z.Li (1 sample), *Neomicrocalamus* Keng f. (3 samples), *Phuphanochloa* Sungkaew & Teerawat (1 sample), and *Temochloa* S.Dransf. (2 samples) were selected as outgroup taxa. Samples are detailed in Suppl. material 1: table SS1.

### Selection of loci for assembly

Four publicly available genomes of the PWB, i.e., *Bonia amplexicaulis* (L.C.Chia, H.L.Fung & Y.L.Yang) N.H.Xia, *Dendrocalamus latiflorus* Munro, *Dendrocalamus sinicus* L.C.Chia & J.L.Sun, *Melocanna baccifera* (Roxb.) Kurz were retrieved for nuclear gene selection (Liu et al. 2024b). Syntenic blocks were identified using JCVI v1.1.17 (Tang et al. 2024). Syntenic groups with missing taxa, short length (≤ 300 bp), and high identity (> 0.9) among subgenomic copies were filtered out. The rest of the genes were compiled into a target file for gene assembly following the instructions of Hybpiper v2.1.8 (Johnson et al. 2016).

### DNA sequencing, read processing, and assembly

Silica gel-dried leaves were used for total DNA extraction, library preparation, and paired-end 150 bp sequencing. Sequencing was performed on the DNBSEQ-T7 platform with a sequencing depth of 15–20× coverage. For raw data processing, Fastp v0.21.0 (Chen et al. 2018) was used to filter adapter sequences and low-quality sequences with default parameters. Clean reads were used for subsequent nuclear gene assembly. Nuclear genes of *Dendrocalamus sinicus* were directly retrieved from its genome (Liu et al. 2024b; Ma et al. 2024). Nuclear genes of the remaining 31 samples were assembled with Hybpiper v2.1.8 using the target file made from the selected loci (Johnson et al. 2016). To obtain high-quality sequences and reduce the interference from potential paralogs, we eliminated assembled sequences with potential paralog warnings identified by Hybpiper and those shorter than 300 bp.

### Dataset construction and phylogenetic analysis

PWB is hexaploid, with subgenomes A, B, and C that have a complicated evolutionary history and exhibit various levels of subgenome dominance (Ma et al. 2024). Four datasets, which contain subgenomic copies of A, B, C, and all subgenomic copies combined, were built to reconstruct species’ trees using the coalescent method. To enhance the stability and accuracy of the species tree inference, the data matrices were further filtered. First, we performed alignment using MAFFT v7.526 (Katoh 2002) for each gene individually. Aligned matrices longer than 900 bp and consisting of more than 30 samples were kept in the following phylogenetic analysis. Gene trees were built using RAxML-NG v1.1.0 (Kozlov et al. 2019) with “--model GTR+G --bs-trees 100” parameters. Newick Utilities’ nw_ed was used to collapse clades in gene trees with bootstrap support below 10% to reduce low-support branches that could compromise accuracy (Junier and Zdobnov 2010). ASTRAL v5.7.8 (Zhang et al. 2018) was used to infer species trees based on modified gene trees for each dataset. The congruence between gene trees and the species tree was assessed by PhyParts v.0.0.1 (Smith et al. 2015) and visualized by phypartspiecharts.py (https://github.com/mossmatters/phyloscripts/blob/master/phypartspiecharts).

## Results

### Nuclear gene loci retrieved from DGS data

The data volume of the 31 newly sequenced samples ranged from 19.8 gigabases (Gb) to 53.2 Gb, with an average of 33.8 Gb. After quality control, the clean data ranged from 18.1 Gb to 51 Gb with an average of 32.6 Gb (approximately 15–20× coverage). Q30 values of all samples exceeded 90%, and GC content ranged from 41.3% to 51% (Suppl. material 1: table S2). The syntenic gene set, derived from four PWB species, contained 17,066 genes. More than 82% of genes were retrieved for all samples except for *Pseudobambusa schizostachyoides*, which yielded only 9,617 genes (56%). The number of genes with potential paralog warnings ranged from 90 to 349, except for *P. schizostachyoides*, with no potential paralog genes. After quality control (removing genes shorter than 300 bp and genes with paralog warnings), the number of nuclear genes ranged from 13,080 to 15,721 genes per sample except for *P. schizostachyoides*, which contained 5,255 genes (Suppl. material 1: table S3).

### Data matrices

We constructed four data matrices for phylogenetic reconstruction, comprising gene copies from subgenomes A, B, C, and all subgenomes combined. After filtering out gene matrices with fewer than 31 samples and shorter than 900 bp, genes retrieved for datasets were 3,009 for subgenome A, 2,945 for subgenome B, and 2,957 for subgenome C. Among the three filtered datasets, only 938 genes were present in all three subgenomes. Finally, the four data matrices were constituted from different/all subgenomic copies of the 938 genes. The DNA sequence matrices for genes are available through the Science Data Bank (https://doi.org/10.57760/sciencedb.37246).

### Phylogenetic relationships revealed by syntenic nuclear genes

Interspecific relationships were identical between the subgenome C dataset and the combined dataset, which were slightly different from those of the subgenome A and B datasets. The only differences in the tree of the subgenome A dataset were the phylogenetic position of *Laobambos*, non-monophyly of *Temochloa*, and non-monophyly of *V. darlacensis*, which were not fully supported (LPP < 1). In the subgenome B tree, the potential new species clustered with the clade comprising *V. darlacensis*, *V. ciliata*, and *V. pusilla* (Fig. 2 and Suppl. material 2: figs S1–S3). Therefore, the tree of the combined dataset was selected as the main topology on the basis of its high node supports and general consensus in topologies among the four datasets. In the main tree, apart from the isolated *Phuphanochloa*, two clades were resolved: one clade contained the genera of *Bonia*, *Temochloa*, *Laobambos*, and *Neomicrocalamus*, and the other comprised the BDG complex. In the BDG complex clade, most genera were monophyletic except for *Dendrocalamus* and *Bambusa*. Species of *Vietnamosasa* clustered in a monophyletic clade, which was sister to *Pseudobambusa schizostachyoides*. The putative new species is sister to the remaining species of *Vietnamosasa*. All currently described species were revealed as monophyletic.

PhyParts analysis revealed phylogenetic discordance between gene trees and the ASTRAL species tree (Fig. 2 and Suppl. material 2: figs S1–S3). 199/207/228 out of 938 genes recovered *Vietnamosasa* as a monophyletic group in the subgenome A/B/C dataset, and 634 out of 2814 in the combined dataset. Furthermore, widespread gene trees and the putative species tree conflict were detected at most nodes across these clades.

## Discussion

### Interspecific relationships within *Vietnamosasa*

Our results demonstrated that syntenic nuclear genes provided high resolution for *Vietnamosasa* with multiple individuals sampled from multiple congeneric species, and resolved the phylogenetic relationships with its closely related genera. *Vietnamosasa* is monophyletic, as revealed in the previous study using *Mi*ddRAD data (Liu et al. 2024a). Three out of four phylogenetic species trees revealed that *V. sakonnakhonensis* diverged first, followed by an unidentified species. *V. darlacensis* and *V. pusilla* are sister to each other, forming a clade sister to *V. ciliata* (Fig. 2 and Suppl. material 2: figs S1, S3). The results of syntenic genes are mostly consistent with the morphology-based taxonomy of *Vietnamosasa*. Morphologically, the three species *V. ciliata*, *V. darlacensis*, and *V. pusilla* bear deciduous culm sheath auricles with oral setae, whereas *V. sakonnakhonensis* has persistent culm sheath auricles without oral setae. Both *V. darlacensis* and *V. pusilla* have a mucronate palea apex, whereas *V. ciliata* has an obtuse palea apex. In addition, *V. pusilla* has longer pseudospikelets (> 5 cm) and glabrous flowering branches. In contrast, *V. darlacensis* has shorter pseudospikelets (< 5 cm), and the flowering branches are covered with white powder. Samples of *V. ciliata* and *V. pusilla* each form a monophyletic clade with high support (LPP = 1) in all four datasets. The only exception is the minor topological discordances in the subgenome A tree (Suppl. material 2: fig. S1) in which *V. darlacensis* is paraphyletic. This phenomenon may result from complicated evolutionary history, requiring additional population genetics studies on these species (Goh et al. 2013; Liu et al. 2020). The unidentified individual CIF92 formed a distinct clade in all species trees. Its grass-mound appearance and long lanceolate foliage blades allow us to confirm its membership within *Vietnamosasa*, despite the limited morphological traits, and our results show its independent taxonomic status in *Vietnamosasa* and relatively greater genetic distance from the three described species, supported by longer internal branch length. However, this species needs to be critically evaluated with morphological and molecular evidence. An interesting feature of CIF92 is that most florets bear one stigma (C.I. Fomichev, D.D. Sokoloff, pers. obs.). In this character, the specimen resembles *V. sakonnakhonensis*. In general, our data allow for reconsideration of the existing view that currently recognized species of *Vietnamosasa* always have three stigmas, which is important in terms of the evolution of stigma number, a question of general importance with respect to the floral evolution of grasses (Sokoloff et al. 2022). It would be very interesting to learn whether the character state of three stigmas is plesiomorphic or apomorphic in *Vietnamosasa* through comparative phylogenetic analysis. Taken together, the taxonomy and evolution of this genus remain to be further explored.

### The new species of *Vietnamosasa*

**Figure 1.**
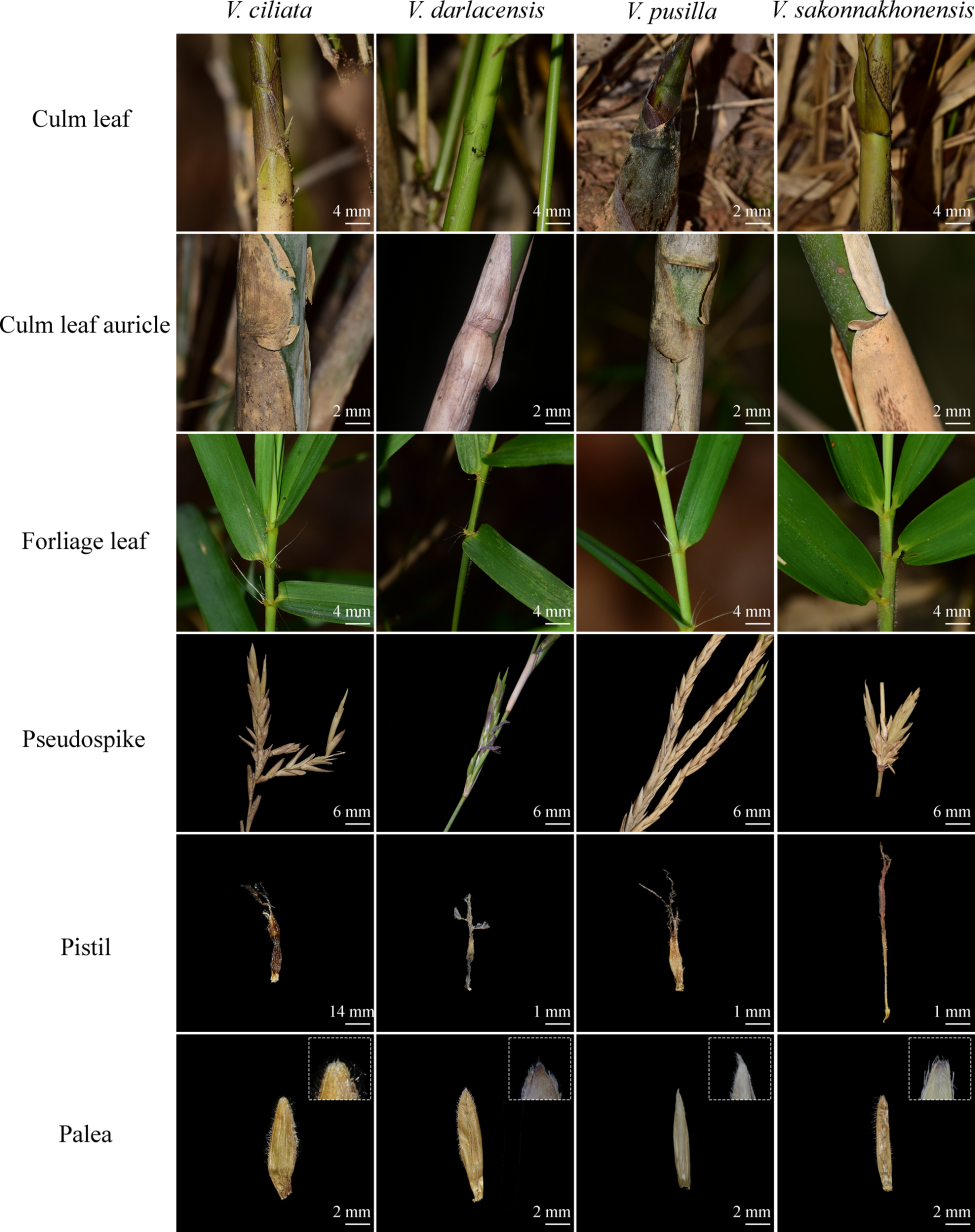
Comparison of morphological characteristics between the potential new species and the other three known species of *Vietnamosasa*, with close-up images showing palea apex.

Both morphological and molecular evidence support the newly collected species as a new species of *Vietnamosasa*. Morphologically, diagnostic characters of the new species fit in the genus *Vietnamosasa* but can be easily distinguished from the other species by both vegetative and inflorescence characters (Table 1). The new species resembles the other three species of *Vietnamosasa* in having long and narrow lanceolate foliage leaves with ciliated auricles, hairy foliage leaf sheaths, indeterminate inflorescences, branches complemented with one dominant branch at the base, and culm leaf sheaths with brown hairs abaxially. However, it can be readily distinguished from the other species by its leaf blade width (1.1–1.3 cm vs. *V. ciliata* 0.5–0.6 cm, *V. darlacensis* 0.4–0.5 cm, *V. pusilla* 0.4–0.8 cm), glabrous (vs. ciliate) margins of the culm leaf sheath, 1 or 2 stigmas (vs. 3), and a longer pistil (10–14 mm vs. *V. ciliata* 4–5 mm, *V. darlacensis* ca. 5 mm, *V. pusilla* ca. 5 mm) (Fig. 1). The culm leaf sheaths of *V. pusilla* and *V. ciliata* are purplish-green when young in the field, which differs from the description of Dransfield (2000). This discrepancy likely stems from two key factors: first, the timing of the survey; second, intraspecific variations of culm leaf sheath color may be influenced by environmental conditions such as light, as differences in sheath color under shade treatments have been reported in other species, e.g., *Phyllostachys violascens* Rivière & C.Rivière and *Pseudosasa amabilis* (McClure) Keng f. (He et al. 2021; Guo and Yu 2023). The glabrous culm leaf sheath margin and the auricles of the culm leaves, while the other three species are ciliate. The middle part of the foliage leaf blade width of the new species is approximately 1.2 cm, wider than the width (4–5 mm) of the other *Vietnamosasa* species. Furthermore, the floret is a key characteristic in bamboo classification. Following the dissection of the florets of the new species, *V. ciliata*, *V. pusilla*, and *V. darlacensis*, we discovered that the length of the pistil and the shape of the top palea can be used to differentiate among the four species; the pistil of the new species is obviously longer than the others; the apices of the palea of *V. ciliata* and *V. sakonnakhonensis* are obtuse, while those of *V. pusilla* and *V. darlacensis* are mucronate. Phylogenetically, *V. sakonnakhonensis* constantly clustered within the *Vietnamosasa* clade with a strong support value (LPP = 1.0), but it was relatively distantly related to the three known species.

**Table 1.** Morphological comparisons among *Vietnamosasa sakonnakhonensis* and three known species of *Vietnamosasa*.

Characters	*V. ciliata*	*V. darlacensis*	*V. pusilla*	*V. sakonnakhonensis*
Culm	More than 2 m tall, 1 cm in diameter	Ca. 1.5 m tall, ca. 1 cm in diameter	0.5–1.5 m tall, 0.3–0.8 cm in diameter	2–2.5 m tall, 1–1.5 cm in diameter
Culm leaf sheath	Ciliate margin, culm surface covered with brown hairs	Ciliate margin, culm surface covered with brown hairs when young and glabrous later	Ciliate margin, culm surface covered with short white fluff and bristles when young, deciduous later	Glabrous margin, abaxial surface covered with brown hairs
Culm leaf auricle	Deciduous with oral setae	Deciduous with oral setae	Absent	Persistent, oral setae absent
Foliage leaf blade	12–19 × 0.5–0.6 cm, pubescent on both surfaces	8–15 × 0.4–0.5 cm, pubescent on both surfaces	12–20 × 0.4–0.8 cm, adaxial pubescent or glabrous, abaxial rarely pubescent	18–22 × 1.1–1.3 cm, pubescent on both surfaces
Flowering branch	Ciliate, 1–4 pseudospikelets per node	Ciliate, 1–5 pseudospikelets per node	Glabrous, 1–3 pseudospikelets per node	Ciliate, 1–3 (–15) pseudospikelets per node
Spikelet	2–4 cm long, 5–9 florets	2–4 cm long, 4–8 florets	3–14 cm long, 7–30 florets	2–2.8 cm long, 3–5 florets
Glume	1, 2	2	1, 2	2, 3
Lemma	Glabrous, 8–11 mm	Glabrous, ca. 9 mm	Glabrous, 8–9 mm	Glabrous, 6–15 mm
Palea	As long as the lemma, apex obtuse, hairy on keels	Subequal or slightly shorter than the lemma, apex mucronate, hairy on keels, margin, and abaxial surface	Slightly longer than the lemma, apex murconate, hairy on keels and abaxial surface	Subequal or slightly longer than the lemma, apex obtuse, hairy on keels, margin, and abaxial surface
Stamen	6, distinct, ca. 4 mm, yellow	6, distinct, 4–5 mm, purple	6, distinct, 3–5 mm, yellow	6, distinct, 5–6 mm, yellow
Pistil	4–5 mm	ca.5 mm	ca. 5 mm	10–14 mm
Stigma	3	3	3	1 or 2

### Species tree discordance

**Figure 2.**
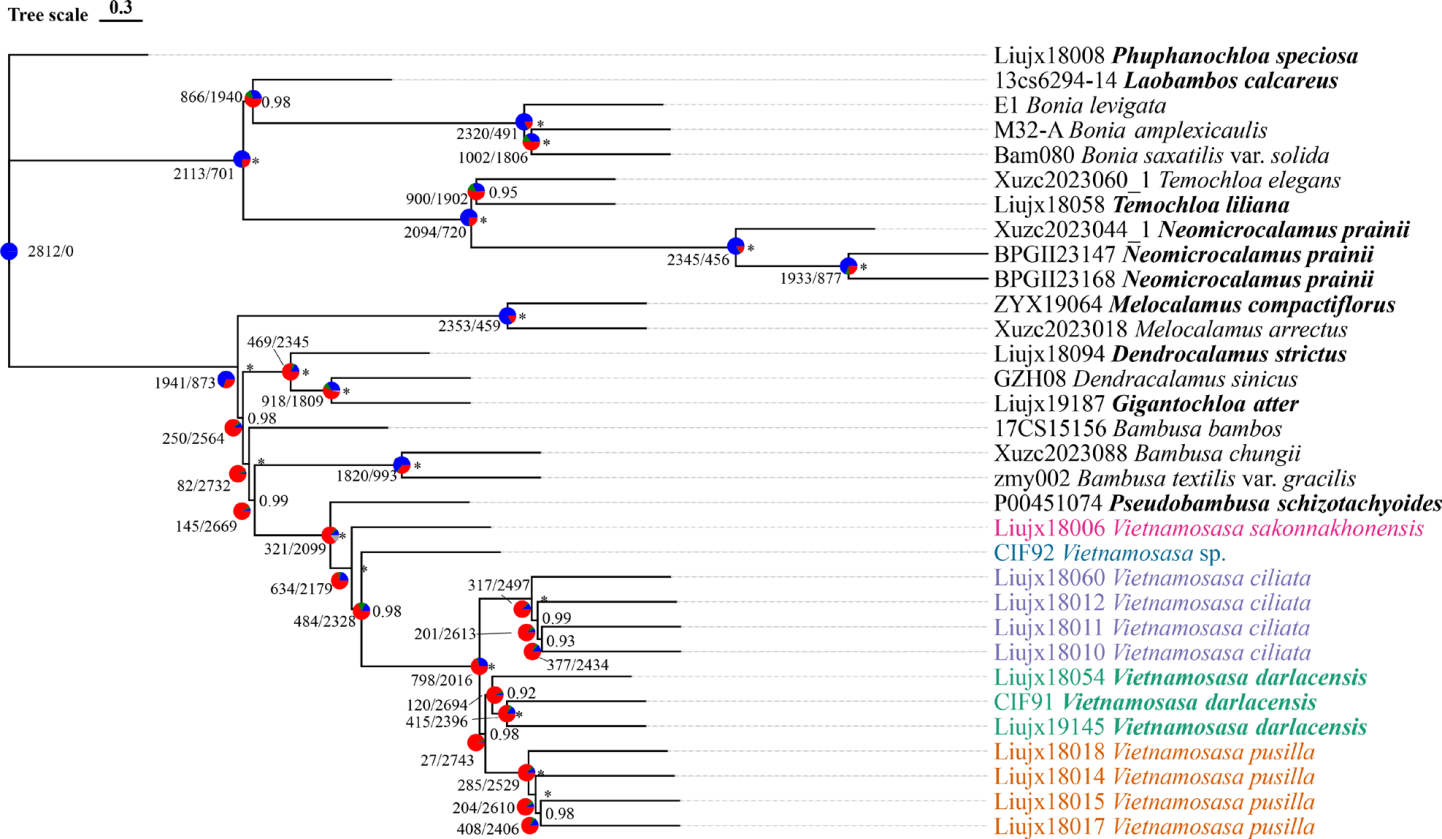
The phylogeny of *Vietnamosasa* with all three subgenomes combined. The symbol * indicates local posterior probability (LPP) equals 1. Types of the generic names are in bold. Numbers near the nodes indicate the quantity of concordant/conflict gene trees. Pie charts at the node present the proportion between gene trees and species tree (blue: support the shown topology, red: all other supported conflict with the shown topology, green: the most common conflict with the shown topology, grey: no information).

We identified four different phylogenetic topologies. The phylogenetic discordance is highest in the subgenome A dataset compared with the other three datasets, particularly involving *Laobambos*, *Temochloa*, and one individual of *V. darlacensis*. PhyParts analysis revealed widespread conflict between gene trees and the species tree inference across all three subgenomes (Fig. 2, Suppl. material 2: figs S1–S3). The red proportion from the pie chart at each node in the species tree indicated the conflict between the gene trees and the species tree inference. This phenomenon is common in bamboos, especially in the BDG complex, likely caused by hybridization and introgression, or incomplete lineage sorting (ILS) (Liu et al. 2020; Guo et al. 2021; Grass Phylogeny Working Group III 2024; Chen et al. 2025a; Niu et al. 2025; Chen et al. 2026). Within the BDG complex clade excluding *Melocalamus*, more than 80% of gene trees were incongruent with the species tree at each node across the three subgenome datasets. Normalized quartet scores of 0.76, 0.77, and 0.77 were estimated for the subgenomes A, B, and C, respectively. The normalized quartet score from the ASTRAL tree roughly estimates the level of ILS. Those relatively high values indicate a consistency among gene trees, implying that ILS is not the dominant cause of the observed discordance.

In the phylogenetic trees of four datasets, *Vietnamosasa* always clusters as monophyletic and is sister to *Pseudobambusa*, which is consistent with the previous study based on *Mi*ddRAD data (Liu et al. 2024a). However, the subgenome A tree revealed critical discordances, including a paraphyly of *Temochloa*, the position of *Laobambos*, and the nesting of a *V. darlacensis* sample within *V. pusilla*. This incongruence indicates that the subgenome A data may contain a complicated evolutionary history. Regardless of tree congruences, *Vietnamosasa* is still monophyletic, and the trees still support the new species status. Resolving the conflicts’ signals requires a more comprehensive sampling across the BDG complex, along with more sophisticated discordance analysis.

### Taxonomic implications of *Pseudobambusa*

*Pseudobambusa* was established by Nguyen (1991), with *P. schizostachyoides* (Kurz) T.Q.Nguyen as the type. However, the validity of this genus is still debated (Ohrnberger 1999; Vorontsova et al. 2016; Soreng et al. 2017). Das et al. (2007) used 32 morphological characteristics and 120 polymorphic alleles to demonstrate that *P. schizostachyoides* was embedded in *Bambusa*, *Dendrocalamus*, and *Gigantochloa*. In our study, despite fewer nuclear genes assembled for *P. schizostachyoides*, phylogenetic results resolved *P. schizostachyoides* as a sister species of the *Vietnamosasa* clade with high support value (LPP = 1) but shorter internal branch length in all species trees, indicating its closest affinity to *Vietnamosasa*. *Pseudobambusa* also has similar morphological characters with *Vietnamosasa*, e.g., exceptionally long lanceolate leaves with ciliate leaf sheaths, and slightly narrowed bases of the culm leaf blades. Furthermore, both *Pseudobambusa* and *Vietnamosasa* are distributed in southern Vietnam. All the aforementioned evidence suggests that *Pseudobambusa* is closely related to, and may be treated as a synonym of *Vietnamosasa*, rather than *Bambusa*, as suggested by Vorontsova et al. (2016) and Soreng et al. (2022). However, the specimen and the original description of *Pseudobambusa* provided limited information. For this study, we were only able to sample a single specimen collected in 1966, without other materials from the field. This specimen contained only one foliage branch, one flowering branch, and several foliage leaves. The original description provides no information on vegetative characters such as the size, leaf shape, culm leaf, and culm features or rhizome structures, except for indicating a woody bamboo with 1–4 subequal branches. For the characters of florets, while the original description provides several key traits like the spikelets subtended by bracts, 3–4 florets per spikelet, lemma longer than palea, 6 stamens, 3 stigmas with narrow-elliptical ovary, and hard caryopsis, it lacks critical details on the inflorescence type, bract, lemma, and palea morphology. Therefore, further research and fieldwork are required to acquire comprehensive data on *Pseudobambusa* to confirm its close relationship with *Vietnamosasa*.

### Key to the species of *Vietnamosasa*

**Table:** 

1	Foliage blade width 1.1–1.3 cm; culm leaf auricles persistent, nearly elliptical to narrowly falcate, oral setae absent; pistil ca. 1–1.4 cm; stigmas 1 or 2	***V. sakonnakhonensis***
–	Foliage blade width ca. 0.4–0.8 cm; culm leaf auricles deciduous, elliptical, oral setae persist; pistil ca. 0.5 cm; stigmas 3	**2**
2	Pseudospikelets longer than 5 cm; flowering branch glabrous	***V. pusilla***
–	Pseudospikelets shorter than 5 cm; flowering branch with white powder	**3**
3	Palea apex obtuse, lemma subequal to the palea	***V. ciliata***
–	Palea apex mucronate, lemma shorter than the pale	***V. darlacensis***

## Taxonomic treatment

### 1. Vietnamosasa ciliata

Taxon classificationPlantaePoalesPoaceae

(A.Camus) T.Q.Nguyen

48DC16F3-AA48-5F7E-9D0D-7C60EDB7729D

Figs 1, 3

Vietnamosasa ciliata (A.Camus) T.Q.Nguyen, Bot. Zhurn. (Moscow & Leningrad) 75(2): 222 (1990).
≡ Arundinaria ciliata A.Camus, Bull. Mus. Natl. Hist. Nat. 25: 672 (1919).
≡ Oreiostachys ciliata (A.Camus) Nakai, J. Arnold Arbor. 6: 152 (1925). (‘*Oreostachys*’)
≡ Racemobambos ciliata (A.Camus) C.S.Chao & Renvoize, Kew Bull. 44: 365 (1989).
≡ Neomicrocalamus ciliatus (A.Camus) Demoly, Bambou Bull. Liais. A. E. B. 21: 14 (1995).

#### Type.

Cambodia. • Compong Thom [Kampong Thom] Province, April 1870; Pierre 6659 (lectotype designated by T. Haevermans in Phytotaxa 137(1): 58, P [digital image!], Barcode: P02581781, isolectotypes designated by T. Haevermans in Phytotaxa 137(1): 58, P [digital image!], Barcode: P02581782, P02581784, P02581785, P02581786).

**Figure 3.**
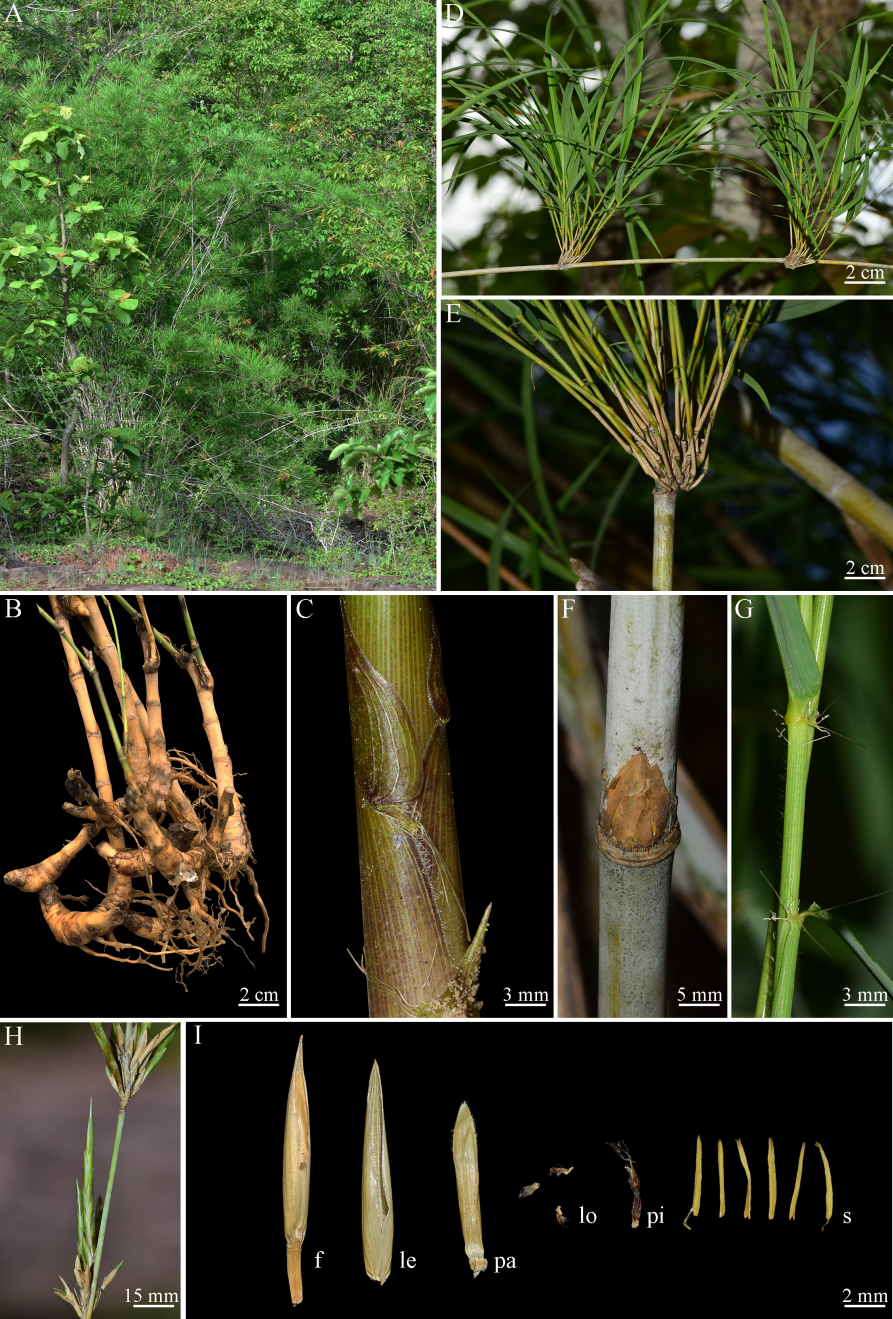
*Vietnamosasa ciliata* (A.Camus) T.Q.Nguyen. **A**. Habitat; **B**. Rhizome; **C**. New shoot; **D, E**. Culm and branch complement; **F**. Node and bud; **G**. Foliage leaf sheath; **H**. Inflorescence; **I**. Fertile floret (f), lemma (le), palea (pa), lodicules (lo), pistil (pi), and stamens (s).

#### Additional specimen examined.

Thailand. • Sakon Nakhon Province: Phanna Nikhom District, Na Nai, 17°22'31.69"N, 103°47'45.44"E, alt. 203 m, 12 June 2018, Jing-Xia Liu, Meng-Yuan Zhou, Jie Liu, Thammarat Boonthammee, Liujx18010 (KUN, Barcode: 1460839, 1460841); ibid., Liujx18011 (KUN, Barcode 1460817, 1460926).

### 2. Vietnamosasa darlacensis

Taxon classificationPlantaePoalesPoaceae

T.Q.Nguyen

55A51F49-A1C9-5344-BAB1-19D6DA8DE59C

Figs 1, 4

Vietnamosasa darlacensis T.Q.Nguyen Bot. Zhurn. (Moscow & Leningrad) 75(2): 222 (1990).

#### Type.

Vietnam. • Dak Nong Province: Yok Don, 12°52'49"N, 107°48'01"E, alt. 200 m, 30 May 2012, My Hanh DIEP 285 (neotype designated by T. Haevermans in Phytotaxa 137(1): 58, P [digital image!], Barcode: P02280067; isoneotypes designated by T. Haevermans in Phytotaxa 137(1): 58, P [digital image!], Barcode: P02280063, P02280064, P02280065, P02280066, P02280068, P02280069; K; KUN! Barcode: 1248538, 1248539, 1248540; MO; SING; RUPP; VNM).

**Figure 4.**
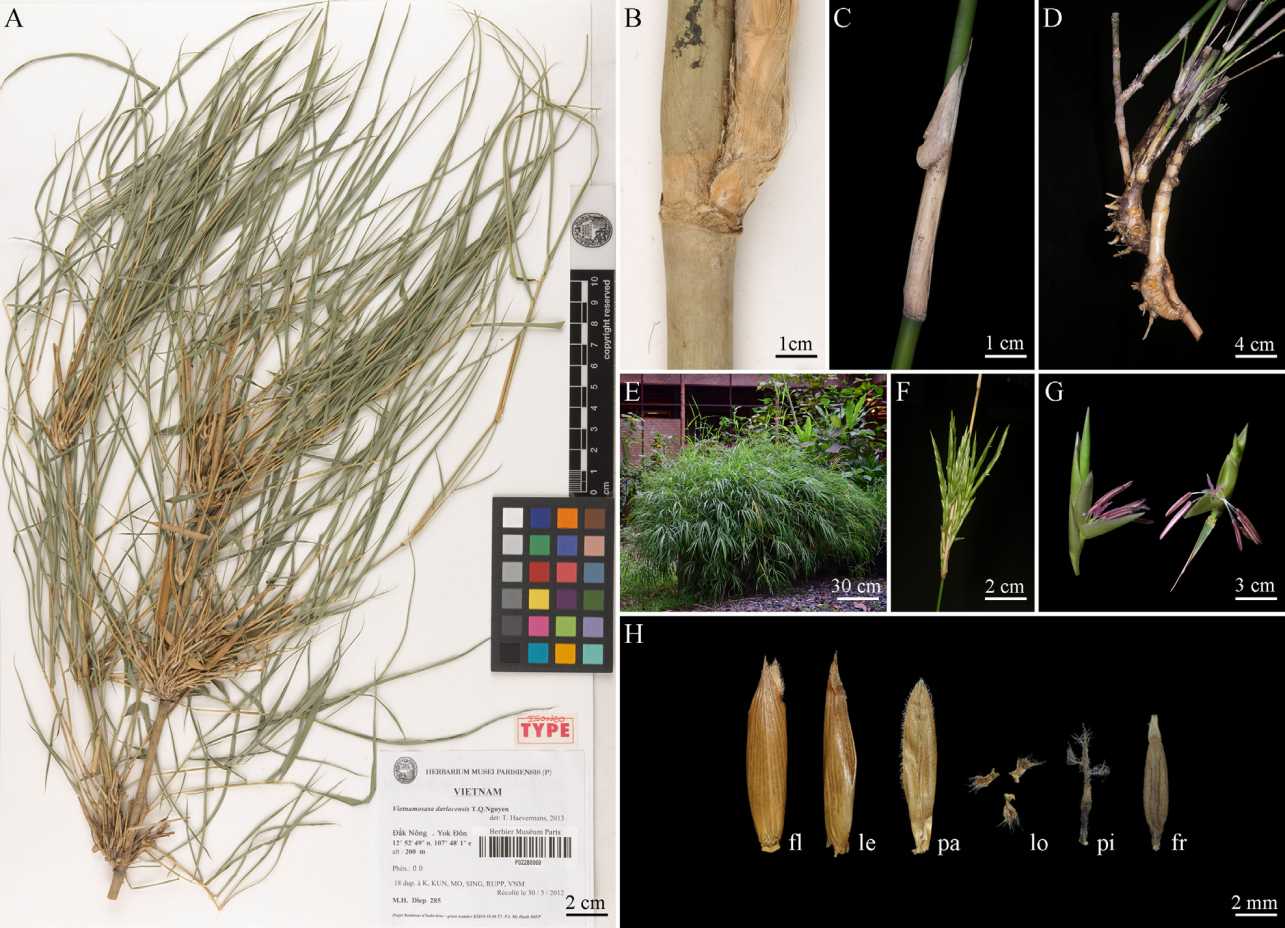
*Vietnamosasa darlacensis* T.Q.Nguyen. **A**. Isoneotype (Barcode: P02280069); **B**. Branch complement (Barcode: P02280063); **C**. Culm sheath; **D**. Rhizome; **E**. Appearance; **F**. Inflorescence branch; **G**. Pseudospikelet; **H**. Fertile floret (fl), lemma (le), palea (pa), lodicules (lo), pistil (pi), and fruit (fr).

### 3. Vietnamosasa pusilla

Taxon classificationPlantaePoalesPoaceae

(A.Chev. & A.Camus) T.Q.Nguyen

6235E8CF-7311-551F-9F49-C8C4088BA150

Figs 1, 5

Vietnamosasa pusilla (A.Chev. & A.Camus) T.Q.Nguyen, Bot. Zhurn. (Moscow & Leningrad) 75(2): 222 (1990).
≡ Arundinaria pusilla A.Chev. & A.Camus, Bull. Mus. Natl. Hist. Nat. 6: 450 (1921).
≡ Chimonobambusa pusilla (A.Chev. & A.Camus) Nakai, J. Arnold Arbor. 6: 151 (1925). (‘*pumila*’)
≡ Neomicrocalamus pusillus (A.Chev. & A.Camus) Demoly, Bambou Bull. Liais. A. E. B. 21: 14 (1995).

#### Type.

Vietnam. • Annam, Lâm Đồng Province: Dran, Lang Bian, elev. 1000–1200 m, A. Chevalier 40600 (lectotype designated by T. Haevermans in Phytotaxa 137(1): 59, P [digital image!], Barcode: P02581779).

**Figure 5.**
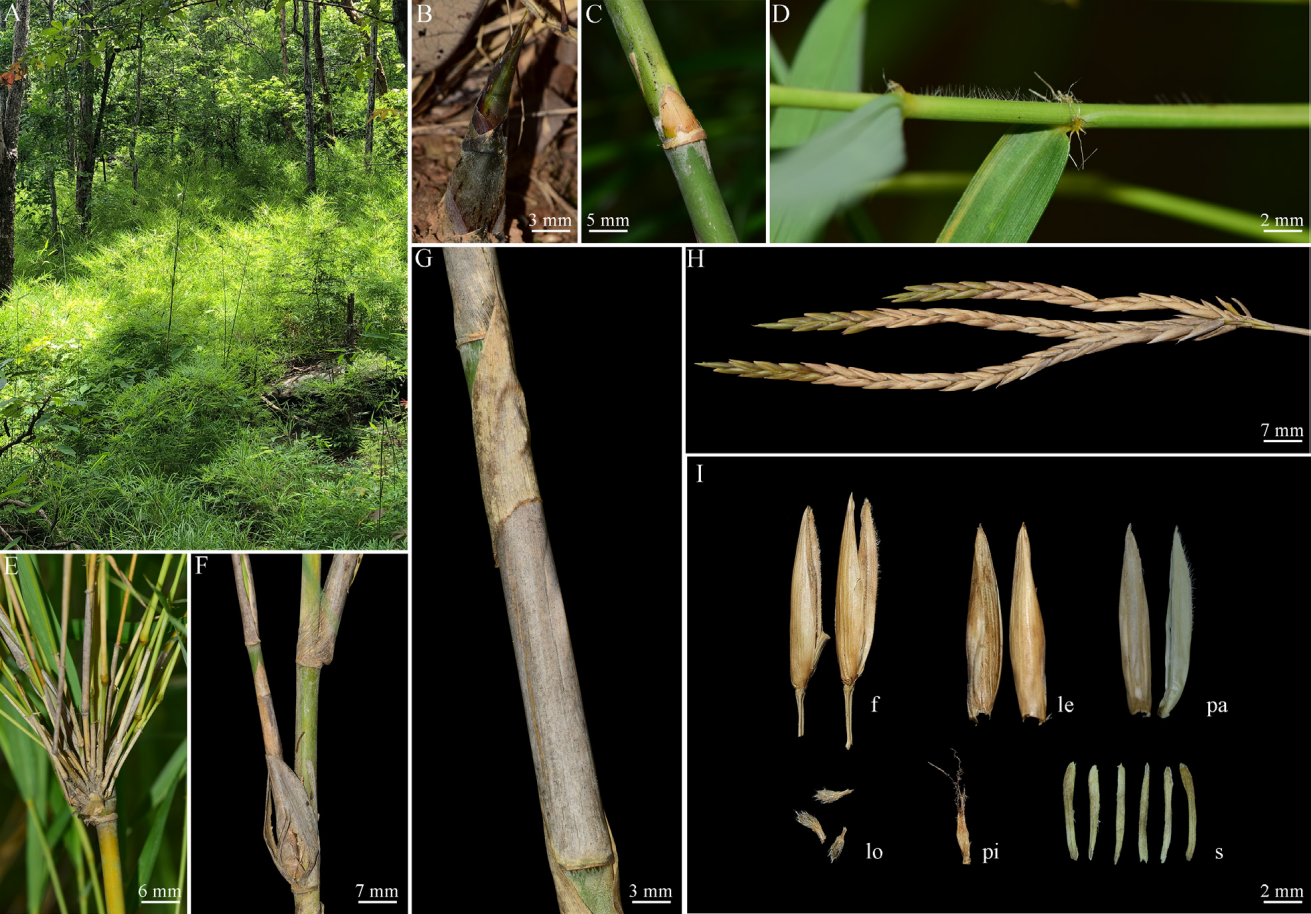
*Vietnamosasa pusilla* (A.Chev. & A.Camus) T.Q.Nguyen. **A**. Habitat; **B**. New shoot; **C**. Node; **D**. Foliage leaf sheath; **E, F**. Culm and branch complement; **G**. Culm with culm leaf; **H**. Inflorescence branch; **I**. Fertile floret (f), lemma (le), palea (pa), lodicules (lo), pistil (pi), and stamens (s).

#### Note.

Nakai transferred *Arundinaria pusilla* to *Chimonobambusa pusilla*, based on *A.* ‘*pumila*’, with an erroneous spelling *A. pusilla*, and published the name as ‘*C. pumila*’ (Nakai 1925). However, *A. pumila* was already a valid name established by Mitford in 1896, which is a heterotypic synonym of *Pleioblastus argenteostriatus* (Regel) Nakai (Freeman-Mitford 1896; Nakai 1933).

#### Additional specimen examined.

Thailand. • Udon Thani Province: Nong Wua So District, Mak Ya, 17°12'3.85"N, 102°38'43.65"E, alt. 292 m, 13 June 2018, Jing-Xia Liu, Meng-Yuan Zhou, Jie Liu, Thammarat Boonthammee, Liujx18014 (KUN, Barcode: 1460832, 1460833); Liujx18015 (KUN, Barcode: 1460447, 1460830); Liujx18018 (KUN, Barcode: 1460788, 1460790).

### 4. Vietnamosasa sakonnakhonensis

Taxon classificationPlantaePoalesPoaceae

D.Z.Li, M.Y.Zhou & X.Feng sp. nov.

64250674-03F5-5B8C-B245-E8D08BB97D73

urn:lsid:ipni.org:names:77378620-1

Figs 1, 6

#### Diagnosis.

The new species resembles *V. ciliata* and *V. darlacensis*, but can be easily distinguished by its glabrous and persistent auricle of the culm leaf, longer pistil (ca. 1 cm), 1–2-lobed hairy stigma, and obtuse apex of the palea.

**Figure 6.**
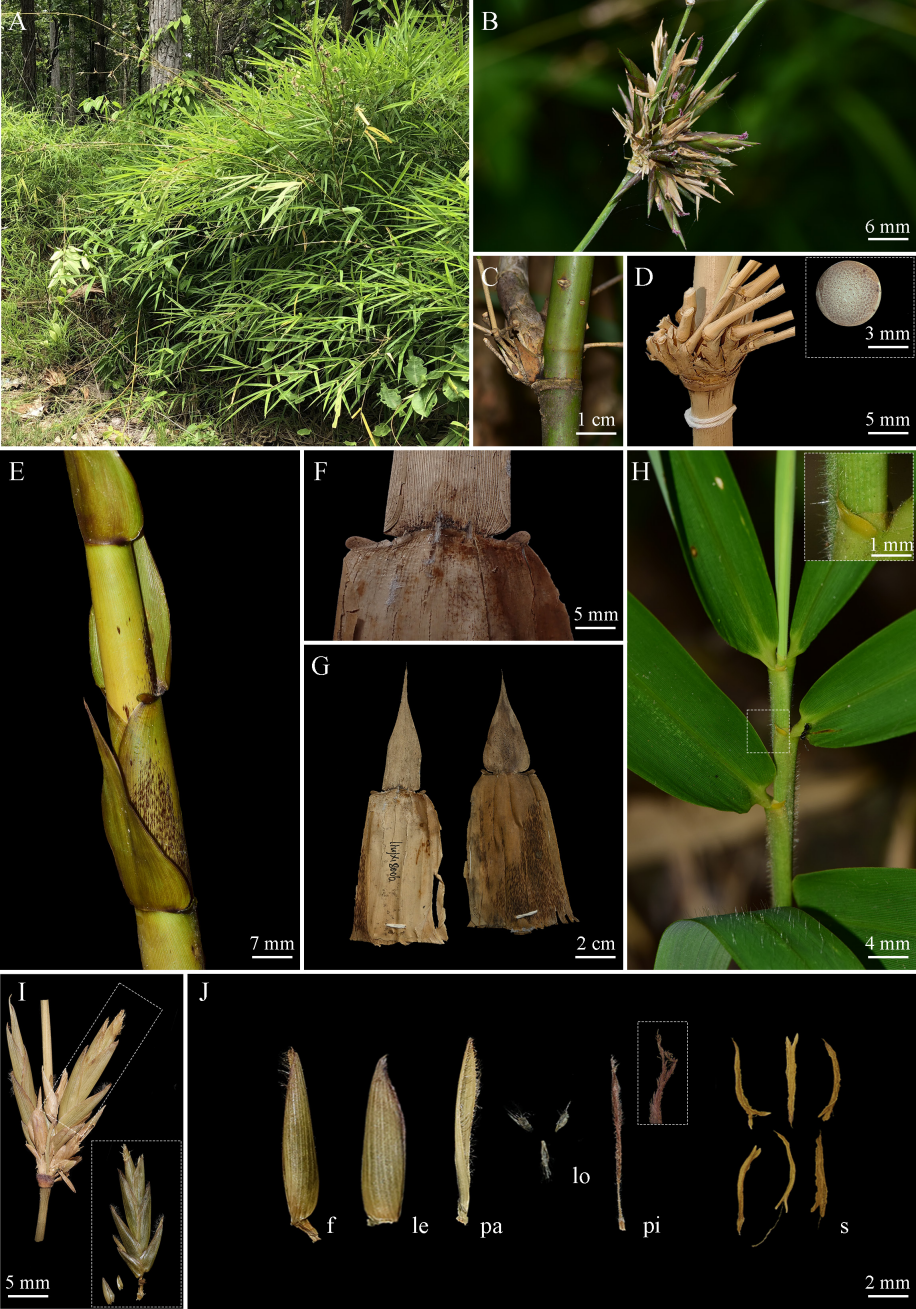
*Vietnamosasa sakonnakhonensis* sp. nov. **A**. Habitat; **B**. Fresh inflorescence; **C, D**. Culm and branch complement, showing transection of culm; **E**. New shoot; **F, G**. Culm leaf; **H**. Foliage leaves, showing foliage leaf auricle and ligule; **I**. Inflorescence, showing pseudospikelet and glumes; **J**. Fertile floret (f), lemma (le), palea (pa), lodicules (lo), pistil (pi), and stamens (s), showing 2 stigmas.

#### Type.

Thailand. • Sakon Nakhon Province: Kut Bak District, Na Mong, 17°04'35.53"N, 103°56'56.09"E, alt. 227 m, 12 June 2018, *Jing-Xia Liu, Meng-Yuan Zhou, Jie Liu, and Thammarat Boonthammee Liujx18006* (holotype, KUN! Barcode 1460823; isotype, KUN! Barcode 1460821, 1460822).

#### Description.

Perennial woody bamboo. Rhizomes pachymorph. Culms apically pendulous, ca. 2–2.5 m tall, 1–1.5 cm in diameter, wall thick or solid; internodes terete, 18–34 cm long, initially sparsely white pubescent, later glabrous; nodes prominent; sheath scars conspicuous. Branches several to many, one dominant branch nearly equal to the main culm in size, along with several slender subequal branches. Culm leaves tardily deciduous, shorter than internodes, 6–8 cm long, 3.5–6 cm wide at base, leathery, thin and brittle, yellow-green initially then becoming yellow before falling off; sheaths densely covered by dark brown bristles abaxially, margin glabrous, apex truncate; auricles persistent, nearly elliptical to narrowly falcate, hard, brown,1.5–2 mm wide, oral setae absent; ligules ca. 1 mm tall, apex denticulate; blades erect, ovate-triangular, apex acuminate, cordate at base, 4–6.5 cm long, glabrous. Foliage leaves 6–8 per ultimate branch; leaf sheaths abaxial and margin white ciliate. Pseudopetioles 1–2 mm long; outer ligules glabrous, ca. 0.5 mm tall; inner ligules lobed, shorter than 1 mm; auricles narrow, orange-yellow, spreading with a few radiated and caducous oral setae; leaf blades long lanceolate, 18–22 × 1.1–1.3 cm, secondary veins 4–6 pairs, adaxial surface white hirsute, abaxial surface pubescent, margins serrulate.

Inflorescence iterauctant, with clusters of 1–3 (–15) fertile pseudospikelets per node; internodes 4–15 cm long, covered with white hair. Pseudospikelets ovate-lanceolate, top and edge purple-green, 20–28 × 2–5 mm; 3–5 florets in each pseudospikelet with the terminal floret sterile; rachilla internodes 0.5–3 mm long, usually distinct and disarticulating with florets, falling separately. Glumes 2 or 3, ovate, 2–4.5 mm long, glabrous, veined. Fertile lemma lanceolate, thinly leathery, green, with top and edge purple, 6–15 mm long, glabrous, apex mucronate. Palea longer than or subequal to lemma, membranous, 2-keeled, apex obtuse, keels and margins with long white cilia, apex margins pubescent. Lodicules 3, 2–3 mm long, membranous, margin with long cilia. Stamens 6, yellow, 5–6 mm long, filaments free. Pistil 1, style 1–1.4 cm long; stigmas 1 or 2, purple, plumose; ovary ovate. Caryopsis unknown.

#### Phenology.

New shoots are produced from May to September. The plants were found flowering at the time of the fieldwork on 12 June 2018.

#### Etymology.

The specific epithet refers to the native distribution in the province of Sakon Nakhon, Thailand.

#### Distribution and habitat.

This species occurs in Kut Bak District, Sakon Nakhon Province, along the roadside and forest edges.

## Supplementary Material

XML Treatment for Vietnamosasa ciliata

XML Treatment for Vietnamosasa darlacensis

XML Treatment for Vietnamosasa pusilla

XML Treatment for Vietnamosasa sakonnakhonensis

## Additional information

### Artificial Intelligence (AI) use

The following AI tools were used in the preparation of this manuscript (select all that apply):

Description: AI was used exclusively for language editing.

### Author ORCIDs

X. Feng https://orcid.org/0009-0007-8430-2100

Z.-C. Xu https://orcid.org/0000-0001-7431-1061

J.-X. Liu https://orcid.org/0000-0003-4205-6400

M. Chen https://orcid.org/0009-0004-0802-3669

D.D. Sokoloff https://orcid.org/0000-0001-6314-9767

C.I. Fomichev https://orcid.org/0000-0001-7590-2103

M.-Y. Zhou https://orcid.org/0000-0003-1492-8494

D.-Z. Li https://orcid.org/0000-0002-4990-724X

### Data availability

All of the data that support the findings of this study are available in the main text or Supplementary Information.

## Supplementary materials

Supplementary material 1 Additional information Data type xlsx Explanation note **table SS1**. Voucher information and sources of samples. **table S2**. The information of sequencing data. **table S3**. Assembly results of PWB syntenic nuclear genes. https://binary.pensoft.net/file/1594984This dataset is made available under the Open Database License (http://opendatacommons.org/licenses/odbl/1.0/). The Open Database License (ODbL) is a license agreement intended to allow users to freely share, modify, and use this Dataset while maintaining this same freedom for others, provided that the original source and author(s) are credited. Xiao Feng, Zu-Chang Xu, Jing-Xia Liu, Mei Chen, Dmitry D. Sokoloff, Constantin I. Fomichev, Meng-Yuan Zhou, De-Zhu Li

Supplementary material 2 Phylogenetic relationships based on subgenome dataset Data type docx Explanation note **fig. S1**. Phylogenetic relationships based on the subgenome A dataset. **fig. S2**. Phylogenetic relationships based on the subgenome B dataset. **fig. S3**. Phylogenetic relationships based on the subgenome C dataset. https://binary.pensoft.net/file/1594985This dataset is made available under the Open Database License (http://opendatacommons.org/licenses/odbl/1.0/). The Open Database License (ODbL) is a license agreement intended to allow users to freely share, modify, and use this Dataset while maintaining this same freedom for others, provided that the original source and author(s) are credited. Xiao Feng, Zu-Chang Xu, Jing-Xia Liu, Mei Chen, Dmitry D. Sokoloff, Constantin I. Fomichev, Meng-Yuan Zhou, De-Zhu Li
