# Supplementary material for: Phylogeny of Vietnamosasa (Poaceae, Bambusoideae) based on syntenic nuclear genes with description of a new species
Source: PhytoKeys. 2026 Apr 14;273:71–91. doi: 10.3897/phytokeys.273.182677 (PMC13100663; doi:10.3897/phytokeys.273.182677)
Supplement: Supplementary material 2 — Phylogenetic relationships based on subgenome dataset [file phytokeys-273-071_article-182677__-s002.docx]

**Supplementary file 1**

**Table S1.** Voucher information and sources of samples

| No. | Taxon | Voucher | Locality |
| --- | --- | --- | --- |
| 1 | *Bambusa bambos* (L.) Voss | 17CS15156 | Mandalay, Myanmar |
| 2 | *Bambusa chungii* McClure | Xuzc2023088 | Guangxi, China |
| 3 | *Bambusa textilis* var. *gracilis* McClure | zmy002 | Guangdong, China |
| 4 | *Bonia amplexicaulis* (L.C.Chia, H.L.Fung & Y.L.Yang) N.H.Xia | M32-A | Hainan, China |
| 5 | *Bonia levigata* (L.C.Chia, H.L.Fung & Y.L.Yang) N.H.Xia | E1 | Guangxi, China |
| 6 | *Bonia saxatilis* var. *solida* (C.D.Chu & C.S.Chao) D.Z.Li | Bam080 | Guangxi, China |
| 7 | *Dendrocalamus sinicus* L.C.Chia & J.L.Sun | GZH08 | Yunnan, China |
| 8 | *Dendrocalamus strictus* (Roxburgh) Nees | Liujx18094 | Thapaikgin, Myanmar |
| 9 | *Gigantochloa atter* (Hassk.) Kurz | Liujx19187 | Jawa, Indonesia |
| 10 | *Laobambos calcareus* Haev., Lamxay & D.Z.Li | 13cs6294-14 | Kham Mouan, Laos |
| 11 | *Melocalamus arrectu*s T.P.Yi | Xuzc2023018 | Yunnan, China |
| 12 | *Melocalamus compactifloru*s (Kurz) Benth. | ZYX19064 | Phu Soi Dao, Thailand |
| 13 | *Neomicrocalamus prainii* (Gamble) Keng f. | BPGII23147 | Xizang, China |
| 14 | *Neomicrocalamus prainii* (Gamble) Keng f. | BPGII23168 | Xizang, China |
| 15 | *Neomicrocalamus prainii* (Gamble) Keng f. | Xuzc2023044_1 | Yunnan, China |
| 16 | *Phuphanochloa speciosa* Sungkaew & Teerawat. | Liujx18008 | Sakon Nakhon, Thailand |
| 17 | *Pseudobambusa schizostachyoides* (Kurz) T.Q.Nguyen | P00451074 | Annam, Vietnam |
| 18 | *Temochloa elegans* N.H.Xia, You Y.Zhang, Z.Y.Cai & Y.H.Tong | Xuzc2023060_1 | Guangxi, China |
| 19 | *Temochloa liliana* S.Dransf. | Liujx18058 | Surat Thani, Thailand |
| 20 | *Vietnamosasa ciliata* (A.Camus) T.Q.Nguyen | Liujx18010 | Sakon Nakhon, Thailand |
| 21 | *Vietnamosasa ciliata* (A.Camus) T.Q.Nguyen | Liujx18011 | Sakon Nakhon, Thailand |
| 22 | *Vietnamosasa ciliata* (A.Camus) T.Q.Nguyen | Liujx18012 | Sakon Nakhon, Thailand |
| 23 | *Vietnamosasa ciliata* (A.Camus) T.Q.Nguyen | Liujx18060 | Udon Thani, Thailand |
| 24 | *Vietnamosasa darlacensis* T.Q.Nguyen | CIF91 | Ninh Thuan, Vietnam |
| 25 | *Vietnamosasa darlacensis* T.Q.Nguyen | Liujx18054 | Chiang Mai, Thailand |
| 26 | *Vietnamosasa darlacensis* T.Q.Nguyen | Liujx19145 | Singapore |
| 27 | *Vietnamosasa pusilla* (A.Chev. & A.Camus) T.Q.Nguyen | Liujx18014 | Udon Thani, Thailand |
| 28 | *Vietnamosasa pusilla* (A.Chev. & A.Camus) T.Q.Nguyen | Liujx18015 | Udon Thani, Thailand |

**Table S1.** Voucher information and sources of samples (continued)

| No. | Taxon | Voucher | Locality |
| --- | --- | --- | --- |
| 29 | *Vietnamosasa pusilla* (A.Chev. & A.Camus) T.Q.Nguyen | Liujx18017 | Udon Thani, Thailand |
| 30 | *Vietnamosasa pusilla* (A.Chev. & A.Camus) T.Q.Nguyen | Liujx18018 | Udon Thani, Thailand |
| 31 | *Vietnamosasa sakonnakhonensis* D.Z.Li, M.Y.Zhou & X.Feng | Liujx18006 | Sakon Nakhon, Thailand |
| 32 | *Vietnamosasa* sp. | CIF92 | Ninh Thuan, Vietnam |

**Table S2.** The information of sequencing data

| Voucher | Taxon | Raw reads | Clean reads | Raw base (bp) | Clean base (bp) | Q30 | GC content |
| --- | --- | --- | --- | --- | --- | --- | --- |
| 17cs15156 | *Bambusa bambos* (L.) Voss | 354,994,122 | 354,501,608 | 53,249,118,300 | 49,409,805,752 | 0.97 | 0.45 |
| Xuzc2023088 | *Bambusa chungii* McClure | 165,677,810 | 165,604,282 | 24,851,671,500 | 23,267,410,390 | 0.97 | 0.46 |
| zmy002 | *Bambusa textilis* var. *gracilis* McClure | 198,422,656 | 198,335,616 | 29,763,398,400 | 27,127,569,680 | 0.97 | 0.46 |
| M32-A | *Bonia amplexicaulis* (L.C.Chia, H.L.Fung & Y.L.Yang) N.H.Xia | 308,478,630 | 308,474,904 | 46,271,794,500 | 45,555,363,235 | 0.98 | 0.42 |
| E1 | *Bonia levigata* (L.C.Chia, H.L.Fung & Y.L.Yang) N.H.Xia | 230,225,152 | 230,223,584 | 34,533,772,800 | 34,425,145,619 | 0.98 | 0.43 |
| Bam080 | *Bonia saxatilis* var. *solida* (C.D.Chu & C.S.Chao) D.Z.Li | 135,681,160 | 134,852,142 | 20,352,174,000 | 20,174,370,060 | 0.94 | 0.44 |
| Liujx18094 | *Dendrocalamus strictus* (Roxburgh) Nees | 229,838,244 | 229,706,688 | 34,475,736,600 | 33,825,147,395 | 0.95 | 0.48 |
| Liujx19187 | *Gigantochloa atter* (Hassk.) Kurz | 221,438,350 | 221,342,492 | 33,215,752,500 | 31,141,853,473 | 0.97 | 0.45 |
| 13cs6294-14 | *Laobambos calcareus* Haev., Lamxay & D.Z.Li | 171,513,942 | 171,437,544 | 25,727,091,300 | 23,244,578,858 | 0.97 | 0.45 |
| Xuzc2023018 | *Melocalamus arrectus* T.P.Yi | 340,742,412 | 340,622,134 | 51,111,361,800 | 50,953,795,756 | 0.98 | 0.42 |
| ZYX19064 | *Melocalamus compactiflorus* (Kurz) Benth. | 199,636,918 | 199,322,278 | 29,945,537,700 | 29,505,284,975 | 0.95 | 0.45 |
| BPGII23147 | *Neomicrocalamus prainii* (Gamble) Keng f. | 165,453,962 | 165,281,262 | 24,818,094,300 | 24,586,219,453 | 0.95 | 0.44 |
| BPGII23168 | *Neomicrocalamus prainii* (Gamble) Keng f. | 184,280,636 | 184,089,716 | 27,642,095,400 | 27,217,174,149 | 0.95 | 0.44 |
| Xuzc2023044_1 | *Neomicrocalamus prainii* (Gamble) Keng f. | 132,137,848 | 131,973,382 | 19,820,677,200 | 19,691,334,742 | 0.94 | 0.43 |
| Liujx18008 | *Phuphanochloa speciosa* Sungkaew & Teerawat. | 189,170,926 | 189,085,006 | 28,375,638,900 | 25,749,750,525 | 0.97 | 0.46 |

**Table S2.** The information of sequencing data (continued)

| Voucher | Taxon | Raw reads | Clean reads | Raw base (bp) | Clean base (bp) | Q30 | GC content |
| --- | --- | --- | --- | --- | --- | --- | --- |
| P00451074 | *Pseudobambusa schizostachyoides* (Kurz) T.Q.Nguyen | 204,280,034 | 204,094,186 | 30,642,005,100 | 18,180,514,675 | 0.98 | 0.51 |
| Xuzc2023060_1 | *Temochloa elegans* N.H.Xia, You Y.Zhang, Z.Y.Cai & Y.H.Tong | 137,793,246 | 137,631,684 | 20,668,986,900 | 20,440,856,041 | 0.95 | 0.44 |
| Liujx18058 | *Temochloa liliana* S.Dransf. | 182,499,044 | 182,413,584 | 27,374,856,600 | 25,891,671,786 | 0.97 | 0.45 |
| Liujx18010 | *Vietnamosasa ciliata* (A.Camus) T.Q.Nguyen | 270,478,358 | 270,475,824 | 40,571,753,700 | 40,442,270,730 | 0.96 | 0.42 |
| Liujx18011 | *Vietnamosasa ciliata* (A.Camus) T.Q.Nguyen | 216,681,034 | 216,680,556 | 32,502,155,100 | 32,449,737,386 | 0.97 | 0.42 |
| Liujx18012 | *Vietnamosasa ciliata* (A.Camus) T.Q.Nguyen | 247,312,328 | 247,178,934 | 37,096,849,200 | 36,984,270,839 | 0.97 | 0.42 |
| Liujx18060 | *Vietnamosasa ciliata* (A.Camus) T.Q.Nguyen | 283,293,540 | 283,190,380 | 42,494,031,000 | 42,412,211,168 | 0.96 | 0.43 |
| CIF91 | *Vietnamosasa darlacensis* T.Q.Nguyen | 226,439,224 | 226,438,868 | 33,965,883,600 | 33,782,185,081 | 0.98 | 0.42 |
| Liujx18054 | *Vietnamosasa darlacensis* T.Q.Nguyen | 247,385,888 | 247,268,774 | 37,107,883,200 | 36,971,889,582 | 0.98 | 0.41 |
| Liujx19145 | *Vietnamosasa darlacensis* T.Q.Nguyen | 254,999,088 | 254,877,316 | 38,249,863,200 | 37,990,295,593 | 0.98 | 0.41 |
| Liujx18014 | *Vietnamosasa* pusilla (A.Chev. & A.Camus) T.Q.Nguyen | 247,109,984 | 246,992,778 | 37,066,497,600 | 36,853,420,522 | 0.98 | 0.42 |
| Liujx18015 | *Vietnamosasa* pusilla (A.Chev. & A.Camus) T.Q.Nguyen | 265,737,844 | 265,603,626 | 39,860,676,600 | 39,739,957,113 | 0.97 | 0.42 |
| Liujx18017 | *Vietnamosasa* pusilla (A.Chev. & A.Camus) T.Q.Nguyen | 256,652,986 | 256,510,904 | 38,497,947,900 | 38,372,133,857 | 0.97 | 0.42 |
| Liujx18018 | *Vietnamosasa* pusilla (A.Chev. & A.Camus) T.Q.Nguyen | 256,216,608 | 256,089,406 | 38,432,491,200 | 34,971,290,704 | 0.97 | 0.46 |
| Liujx18006 | *Vietnamosasa sakonnakhonensis* D.Z.Li, M.Y.Zhou & X.Feng | 239,520,956 | 239,386,362 | 35,928,143,400 | 35,782,867,931 | 0.98 | 0.42 |
| CIF92 | *Vietnamosasa* sp. | 219,543,312 | 219,542,514 | 32,931,496,800 | 32,734,365,114 | 0.98 | 0.45 |

**Table S3.** Assembly results of PWB syntenic nuclear genes

| Voucher | Taxon | Genes with seqs | Paralog warnings | Genes length < 300 bp | Genes retained after filtering | Genes for tree building |
| --- | --- | --- | --- | --- | --- | --- |
| 17cs15156 | *Bambusa bambos* (L.) Voss | 16,414 | 276 | 417 | 15,721 | 2,814 |
| Xuzc2023088 | *Bambusa chungii* McClure | 16,128 | 114 | 563 | 15,451 | 2,813 |
| zmy002 | *Bambusa textilis* var. *gracilis* McClure | 16,110 | 107 | 594 | 15,409 | 2,814 |
| M32-A | *Bonia amplexicaulis* (L.C.Chia, H.L.Fung & Y.L.Yang) N.H.Xia | 16,033 | 185 | 561 | 15,287 | 2,812 |
| E1 | *Bonia levigata* (L.C.Chia, H.L.Fung & Y.L.Yang) N.H.Xia | 15,837 | 254 | 491 | 15,092 | 2,811 |
| Bam080 | *Bonia saxatilis* var. *solida* (C.D.Chu & C.S.Chao) D.Z.Li | 15,597 | 179 | 484 | 14,934 | 2,810 |
| GZH08 | *Dendrocalamus sinicus* L.C.Chia & J.L.Sun | 14,439 | / | 5 | 14,435 | 2,728 |
| Liujx18094 | *Dendrocalamus strictus* (Roxburgh) Nees | 16,293 | 262 | 467 | 15,564 | 2,813 |
| Liujx19187 | *Gigantochloa atter* (Hassk.) Kurz | 15,868 | 90 | 653 | 15,125 | 2,813 |
| 13cs6294-14 | *Laobambos calcareus* Haev., Lamxay & D.Z.Li | 15,890 | 118 | 659 | 15,113 | 2,806 |
| Xuzc2023018 | *Melocalamus arrectus* T.P.Yi | 15,928 | 200 | 552 | 15,176 | 2,813 |
| ZYX19064 | *Melocalamus compactiflorus* (Kurz) Benth. | 15,253 | 129 | 774 | 14,350 | 2,813 |
| BPGII23147 | *Neomicrocalamus prainii* (Gamble) Keng f. | 15,560 | 163 | 664 | 14,733 | 2,812 |
| BPGII23168 | *Neomicrocalamus prainii* (Gamble) Keng f. | 15,311 | 160 | 674 | 14,477 | 2,812 |
| Xuzc2023044_1 | *Neomicrocalamus prainii* (Gamble) Keng f. | 14,047 | 111 | 856 | 13,080 | 2,799 |
| Liujx18008 | *Phuphanochloa speciosa* Sungkaew & Teerawat. | 16,299 | 171 | 565 | 15,563 | 2,812 |
| P00451074 | *Pseudobambusa schizostachyoides* (Kurz) T.Q.Nguyen | 9,617 | 0 | 4362 | 5,255 | 1,485 |
| Xuzc2023060_1 | *Temochloa elegans* N.H.Xia, You Y.Zhang, Z.Y.Cai & Y.H.Tong | 15,258 | 160 | 692 | 14,406 | 2,808 |
| Liujx18058 | *Temochloa liliana* S.Dransf. | 15,837 | 140 | 647 | 15,050 | 2,808 |
| Liujx18010 | *Vietnamosasa ciliata* (A.Camus) T.Q.Nguyen | 16,190 | 296 | 476 | 15,418 | 2,811 |

**Table S3.** Assembly results of PWB syntenic nuclear genes (continued)

| Voucher | Taxon | Genes with seqs | Paralog warnings | Genes length < 300 bp | Genes retained after filtering | Genes for tree building |
| --- | --- | --- | --- | --- | --- | --- |
| Liujx18011 | *Vietnamosasa ciliata (A.Camus)* T.Q.Nguyen | 16,067 | 251 | 512 | 15,304 | 2,814 |
| Liujx18012 | *Vietnamosasa ciliata* (A.Camus) T.Q.Nguyen | 16,220 | 292 | 467 | 15,461 | 2,812 |
| Liujx18060 | *Vietnamosasa ciliata* (A.Camus) T.Q.Nguyen | 16,240 | 349 | 445 | 15446 | 2,814 |
| CIF91 | *Vietnamosasa darlacensis* T.Q.Nguyen | 16,074 | 249 | 479 | 15,346 | 2,813 |
| Liujx18054 | *Vietnamosasa darlacensis* T.Q.Nguyen | 16,141 | 237 | 510 | 15,394 | 2,812 |
| Liujx19145 | *Vietnamosasa darlacensis* T.Q.Nguyen | 16,206 | 234 | 490 | 15,482 | 2,812 |
| Liujx18014 | *Vietnamosasa pusilla* (A.Chev. & A.Camus) T.Q.Nguyen | 16,167 | 256 | 503 | 15,408 | 2,814 |
| Liujx18015 | *Vietnamosasa pusilla* (A.Chev. & A.Camus) T.Q.Nguyen | 16,224 | 345 | 450 | 15,429 | 2,814 |
| Liujx18017 | *Vietnamosasa pusilla* (A.Chev. & A.Camus) T.Q.Nguyen | 16,215 | 316 | 460 | 15,439 | 2,814 |
| Liujx18018 | *Vietnamosasa pusilla* (A.Chev. & A.Camus) T.Q.Nguyen | 16,357 | 181 | 512 | 15,664 | 2,814 |
| Liujx18006 | *Vietnamosasa sakonnakhonensis* D.Z.Li, M.Y.Zhou & X.Feng | 16,017 | 204 | 502 | 15,311 | 2,812 |
| CIF92 | *Vietnamosasa* sp. | 14,693 | 130 | 724 | 13,839 | 2,808 |

**Supplementary file 2**


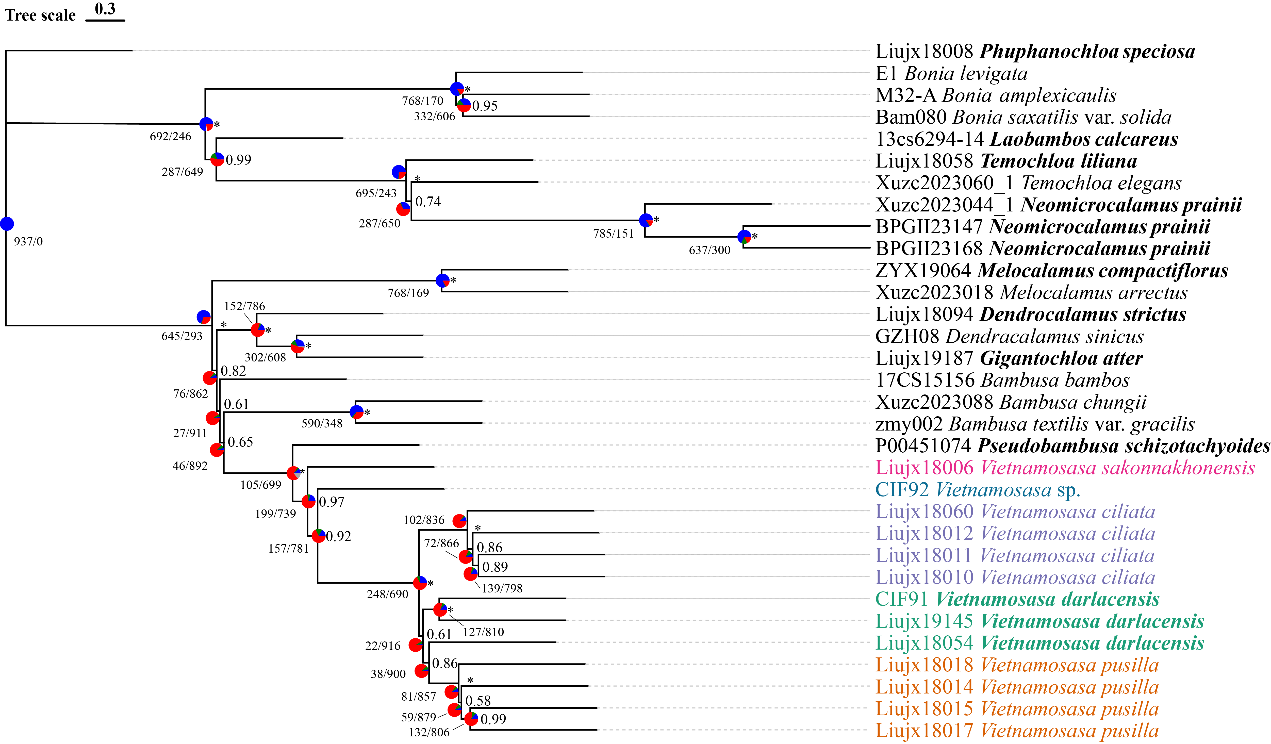


**Figure S1.** Phylogenetic relationships based on the subgenome A dataset. * indicates local posterior probability (LPP) equals 1. Type of the genus is in bold. Numbers near the nodes indicate the quantity of concordant/conflict gene trees. Pie charts at the node represent the proportion of conflicting status between gene trees and species tree (blue: support the shown topology, red: all other supported conflict with the shown topology, green: the most common conflict with the shown topology, grey: no information).


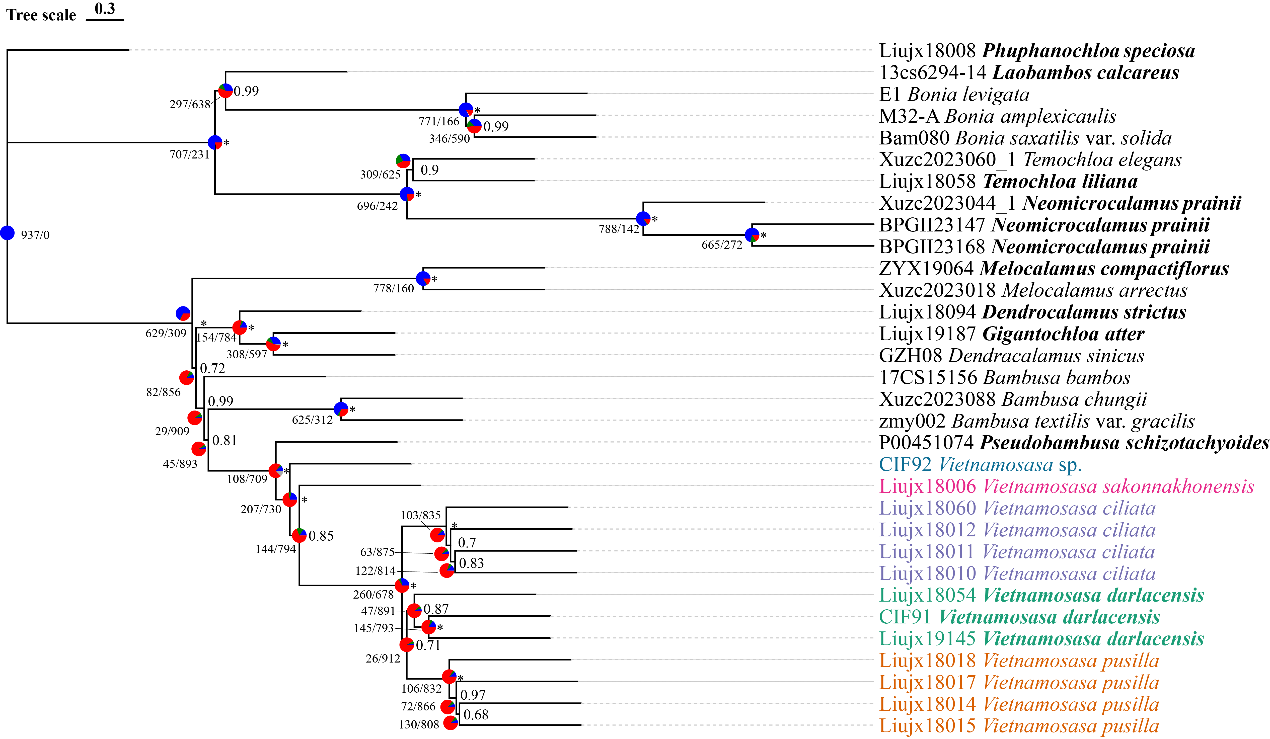


**Figure S2.** Phylogenetic relationships based on the subgenome B dataset. * indicates local posterior probability (LPP) equals 1. Type of the genus is in bold. Numbers near the nodes indicate the quantity of concordant/conflict gene trees. Pie charts at the node represent the proportion of conflicting status between gene trees and species tree (blue: support the shown topology, red: all other supported conflict with the shown topology, green: the most common conflict with the shown topology, grey: no information).


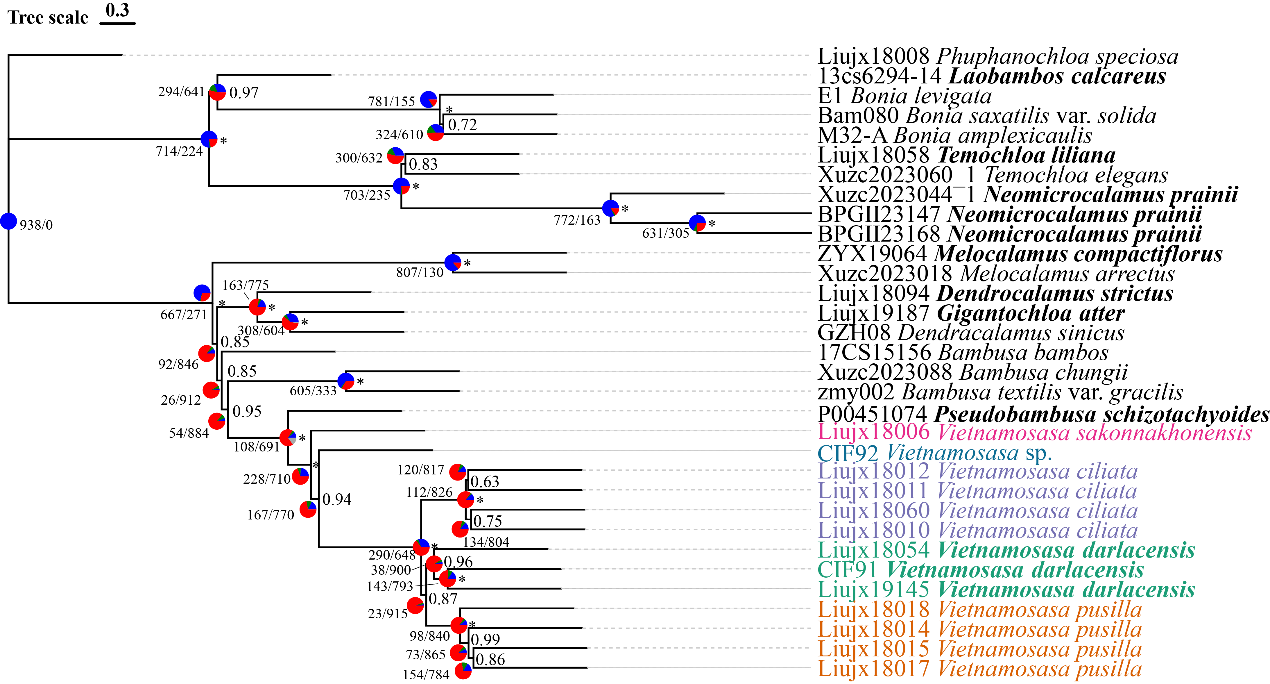


**Figure S3.** Phylogenetic relationships based on the subgenome C dataset. * indicates local posterior probability (LPP) equals 1. Type of the genus is in bold. Numbers near the nodes indicate the quantity of concordant/conflict gene trees. Pie charts at the node present the proportion between gene trees and species tree (blue: support the shown topology, red: all other supported conflict with the shown topology, green: the most common conflict with the shown topology, grey: no information).
